# Sphingosine kinase/sphingosine 1-phosphate axis: a new player for insulin-like growth factor-1-induced myoblast differentiation

**DOI:** 10.1186/2044-5040-2-15

**Published:** 2012-07-12

**Authors:** Caterina Bernacchioni, Francesca Cencetti, Sabrina Blescia, Chiara Donati, Paola Bruni

**Affiliations:** 1 Department of Biochemical Sciences, University of Florence, GB Morgagni 50, 50134, Florence, Italy; 2 Interuniversity Institute of Myology (IIM), Padova, Italy

**Keywords:** IGF-1, Myoblasts, Myogenic differentiation, Sphingosine 1-phosphate, Sphingosine kinase, Sphingosine 1-phospate receptors

## Abstract

**Background:**

Insulin-like growth factor-1 (IGF-1) is the most important physiological regulator of skeletal muscle progenitor cells, which are responsible for adult skeletal muscle regeneration. The ability of IGF-1 to affect multiple aspects of skeletal muscle cell biology such as proliferation, differentiation, survival and motility is well recognized, although the molecular mechanisms implicated in its complex biological action are not fully defined. Since sphingosine 1-phosphate (S1P) has recently emerged as a key player in skeletal muscle regeneration, we investigated the possible involvement of the sphingosine kinase (SK)/S1P receptor axis on the biological effects of IGF-1 in murine myoblasts.

**Methods:**

RNA interference, chemical inhibition and immunofluorescence approaches were used to assess the role of the SK/S1P axis on the myogenic and mitogenic effects of IGF-1 in C2C12 myoblasts.

**Results:**

We show that IGF-1 increases SK activity in mouse myoblasts. The effect of the growth factor does not involve transcriptional regulation of SK1 or SK2, since the protein content of both isoforms is not affected; rather, IGF-1 enhances the fraction of the active form of SK. Moreover, transactivation of the S1P_2_ receptor induced by IGF-1 via SK activation appears to be involved in the myogenic effect of the growth factor. Indeed, the pro-differentiating effect of IGF-1 in myoblasts is impaired when SK activity is pharmacologically inhibited, or SK1 or SK2 are specifically silenced, or the S1P_2_ receptor is downregulated. Furthermore, in this study we show that IGF-1 transactivates S1P_1_/S1P_3_ receptors via SK activation and that this molecular event negatively regulates the mitogenic effect elicited by the growth factor, since the specific silencing of S1P_1_ or S1P_3_ receptors increases cell proliferation induced by IGF-1.

**Conclusions:**

We demonstrate a dual role of the SK/S1P axis in response to myoblast challenge with IGF-1, that likely is important to regulate the biological effect of this growth factor. These findings add new information to the understanding of the mechanism by which IGF-1 regulates skeletal muscle regeneration.

## Background

Sphingosine 1-phosphate (S1P) is a bioactive lipid that is physiologically present in serum and capable of regulating multiple essential cellular processes, including cell growth and survival, cell motility and invasion, angiogenesis, lymphocyte trafficking and immune regulation. S1P is synthesized from sphingosine by a phosphorylation reaction catalyzed by the sphingosine kinases (SKs) SK1 and SK2, which are highly conserved enzymes activated by numerous stimuli [1]. S1P levels are tightly regulated by the balance between biosynthesis catalyzed by SKs, reversible conversion to sphingosine mediated by specific and non-specific lipid phosphatases, and S1P lyase (SPL)-dependent degradation [2,3]. S1P exerts its functions either as a second messenger or as a ligand of five specific G-protein coupled receptors named S1P receptors (S1PR). S1PR are differentially coupled to one or multiple G-proteins and they can activate a variety of signaling pathways determining distinct and even contrasting final cellular effects.

Recently it has emerged that S1P plays a key role in the biology of skeletal muscle progenitor cells [4]. Indeed, the bioactive sphingolipid exerts a strong mitogenic action in satellite cells [5] and also stimulates their cell motility [6], whereas in myoblasts it behaves as pro-differentiating [7] and chemorepellant cue [8]. Our recent findings have demonstrated that the S1P signaling axis, under the control of a number of physiological and pathophysiological extracellular agents such as tumor necrosis factor α (TNFα), platelet-derived growth factor (PDGF) and transforming growth factor β (TGFβ) [9-11], is the mediator of fundamental biological responses evoked by the above mentioned agonists. The obtained results are in keeping with a very complex mechanism of action of S1P that can have both beneficial and unfavorable effects on skeletal muscle regeneration. Indeed, we have demonstrated that endogenous S1P metabolism, stimulated by SK1 activation and S1P_1_ engagement, is critical for myoblast motility and attenuation of the mitogenic response elicited by PDGF [11]; however, we have also shown that the SK/S1P axis is exploited by TGFβ to convey its detrimental pro-fibrotic effect and that upregulation of S1P metabolism mediated by SK1 together with enhanced expression of S1P_3_ account for transdifferentiation of myoblasts into myofibroblasts, which are responsible for deposition of extracellular matrix protein [9].

Insulin-like growth factor 1 (IGF-1) is a hormone peptide that, beside being released into the blood from the liver, can be synthesized by the target tissues, including skeletal muscle, where IGF-1 concentrations are locally regulated. IGF-1 plays a key role in skeletal muscle regeneration as it is able to stimulate myoblast proliferation [12]. Moreover, unlike other growth factors, IGF-1 also stimulates myogenic differentiation and induces myocyte hypertrophy [12], supporting the notion that IGF-1 is a master regulator of skeletal muscle biology. These seemingly contradictory roles of the growth factor highlight the importance of understanding how IGF-1 cooperates with other mitogenic and differentiating stimuli to induce either, or both, these events. In this regard, given the fundamental role of IGF-1 in skeletal muscle and its incompletely defined mechanism of action, we focused on the possible involvement of the SK/S1P axis on the biological effect of the growth factor. Data reported here demonstrate for the first time that IGF-1 activates SKs in mouse myoblasts. The consequent S1P_2_ transactivation appears to be implicated in the myogenic effect of the growth factor. Moreover, the engagement of S1P_1_/S1P_3_ in IGF-1 signaling, via a mechanism dependent on SK activation, inhibits myoblast proliferation, which points to a role for the SK/S1P axis in the control of the biological outcome of IGF-1 in skeletal muscle.

These results highlight a new role for the SK/S1P axis in IGF-1-regulated signaling which is important for the biological response exerted by the growth factor. This could be exploited to improve skeletal muscle regeneration.

## Methods

### Materials

All biochemicals, TRI reagent, cell culture reagents, DMEM, fetal calf serum, protease inhibitor cocktail, monoclonal anti-skeletal fast myosin heavy chain (MHC) (clone MY-32), bovine serum albumin (BSA) were purchased from Sigma-Aldrich (St Louis, MO, USA). Mouse skeletal muscle C2C12 cells were obtained from the American Type Culture Collection (Manassas, VA, USA). Propidium iodide was obtained from Calbiochem (San Diego, CA, USA). Recombinant IGF-1 was obtained from PeproTech (London, UK). SKI-2 [2-(p-hydroxyanilino)-4-(p-chlorophenyl)thiazole] and U0126 were obtained from Calbiochem. siRNA duplexes corresponding to two DNA target sequences of mouse SK1 (5’UAGGAACUGUGGCCUCUAAdTdT3’, 5’GUGUUAUGCAUCUGUUCUAdTdT3’), mouse SK2 (5′GCCUACUUCUGCAUCUACAdTdT3′; 5′CCUCAUACAGACAGAACGAdTdT3′), mouse S1P_1_ (5′UCACCUACUACUGUUAGAdTdT3′; 5′CUUGCUAACUAUUUGGAAAdTdT3′), mouse S1P_2_ (5’CUCUGUACGUCCGAAUGUAdTdT3’, 5’GACUAAUCAGAUUGUAGUAdTdT3’), mouse S1P_3_ (SASI_Mm01_00145232, SASI_Mm01_00145233), mouse S1P_4_ (SASI_Mm01_00094192, SASI_Mm01_00094193), mouse SPL (SASI_Mm01_00122643) and scrambled siRNA (5′UUCUCCGAACGUGUCACGUdTdT3′) were obtained from Sigma-Proligo (The Woodlands, TX, USA). Lipofectamine RNAiMAX was purchased from Invitrogen (Carlsbad, CA, USA). Enhanced chemiluminescence reagents and [γ-^32^P]ATP (3000 Ci/mmol) were obtained from GE Healthcare Europe (Milan, Italy). Secondary antibodies conjugated to horseradish peroxidase, monoclonal anti-β-actin and monoclonal anti-myogenin antibodies were obtained from Santa Cruz Biotechnology (Santa Cruz, CA, USA). Monoclonal anti-caveolin-3 antibodies were from BD Biosciences Transduction Laboratories (Lexington, KY, USA). Specific anti-SK1 polyclonal antibodies (directed against the 16 carboxy-terminal amino acids of the mouse SK1) [13] were kindly provided by Dr Y Banno (Gifu University School of Medicine, Gifu, Japan). Rabbit polyclonal antibodies generated against SK2 [14] were a kind gift from Dr S Nakamura (Department of Molecular and Cellular Biology, Kobe University Graduate School of Medicine, Kobe, Japan). Phospho-human SK1 specific polyclonal antibodies (directed against the phosphopeptide CGSKTPApSPVVVQQ centered around phospho-Ser^225^ of human SK1) [15] were kindly provided by Dr S Pitson (Hanson Institute, institute of Medical and Veterinary Science, Adelaide, Australia). Fluorescein-conjugated horse anti-mouse secondary antibodies were obtained from Vector Laboratories (Burlingame, CA, USA). Specific polyclonal rabbit anti-mouse SPL antibodies (directed against the C-terminal peptide 551-TTDPVTQGNQMNGSPKPR-568) [16] were a kind gift from Dr RL Proia (National Institute of Diabetes and Digestive and Kidney Disease, National Institute of Health, Bethesda, USA). All reagents and probes required to perform real-time PCR were from Applied Biosystems (Foster City, CA, USA). ^3^ H]thymidine (20 Ci/mmol) was from Perkin Elmer (Waltham, MA, USA).

### Cell culture

Murine C2C12 myoblasts were routinely grown in DMEM supplemented with 10% fetal calf serum, 2 mM L-glutamine, 100 U/ml penicillin, and 100 μg/ml streptomycin at 37°C in 5% CO_2_. For myogenic differentiation experiments, cells were seeded in p35 plates, and when 90% confluent they were shifted to DMEM without serum containing 1 mg/ml BSA. For proliferation experiments, cells were seeded in 12-well plates and utilized when approximately 50% confluent.

When requested, cells were incubated with inhibitors 30 minutes before challenge with IGF-1.

### Sphingosine kinase activity assay

SK activity was measured as described by Olivera and colleagues [17] with a few modifications, as described previously [10]. Specific activity of SK was expressed as picomoles of S1P formed per minute per milligram of protein.

### Cell transfection

C2C12 cells were transfected with Lipofectamine RNAiMAX according to the manufacturer's instructions. Briefly, Lipofectamine RNAiMAX was incubated with siRNA in DMEM without serum and antibiotics at room temperature for 20 minutes, and afterwards the lipid/RNA complexes were added with gentle agitation to C2C12 cells to a final concentration of 50 nM in serum containing DMEM. After 24 h, cells were shifted to DMEM without serum containing 1 mg/ml BSA and then used for the experiments within 48 h from the beginning of transfection. The specific gene knockdown was evaluated by Western blot analysis or alternatively by real-time PCR.

### Cellular fractionation

The medium of control and agonist-treated C2C12 cells was removed and the cells were washed twice with ice-cold PBS, scraped, and collected by centrifugation (1000 × g). Cells were dispersed in a buffer solution containing 10 mM HEPES, pH 7.4, 1 mM EGTA, 1 mM EDTA, 250 mM sucrose, 5 mM NaN_3_, and protease inhibitors (1 mM 4-(2-aminoethyl)benzenesulfonyl fluoride, (AEBSF) 0.3 μM aprotinin, 10 μg/ml leupeptin and 10 μg/ml pepstatin) and disrupted in a Dounce homogenizer (100 strokes). Lysates were centrifuged (10 minutes, 800 × g) and the resulting supernatant was centrifuged again at 200,000 × g for 1 h to separate cytosolic and total particulate fractions.

### Western blot analysis

C2C12 cells were lysed for 30 minutes at 4°C in a buffer containing 50 mM Tris, pH 7.5, 120 mM NaCl, 1 mM EDTA, 6 mM EGTA, 15 mM Na_4_P_2_O_7_, 20 mM NaF, 1% Nonidet and protease inhibitor cocktail (1.04 mM AEBSF, 0.08 μM aprotinin, 0.02 mM leupeptin, 0.04 mM bestatin, 15 μM pepstatin A, and 14 μM E-64). To prepare total cell lysates, cell extracts were centrifuged for 15 minutes at 10,000 × g at 4°C. Proteins from cell lysates, cytosolic and membrane fractions were resuspended in Laemmli's SDS sample buffer. Samples were subjected to SDS-PAGE for 90 minutes at 100 mA before transfer of proteins to PVDF membranes. Membranes were incubated overnight with the primary antibodies at 4°C and then with specific secondary antibodies for 1 h at room temperature. Bound antibodies were detected by chemiluminescence.

### Cell immunofluorescence

Cells were seeded on microscope slides, pre-coated with 2% gelatine, and then pre-incubated with SK specific inhibitor (10 μM SKI-2) before being challenged with 50 ng/ml IGF-1. After 72 h cells were fixed in 2% paraformaldehyde in PBS for 20 minutes and permeabilized in 0.1% Triton X-100-PBS for 30 minutes. Cells were then blocked in 3% BSA for 1 h and incubated with anti-MHC antibody for 2 h and fluorescein-conjugated anti-mouse secondary antibody for 1 h. To stain nuclei, the specimen was incubated with 50 μg/ml propidium iodide in PBS for 15 minutes. Images were obtained using a Leica SP5 laser scanning confocal microscope (Leica Microsystems GmbH, Wetzlar, Germany) with a 40× objective. To quantify the differentiation and fusion of C2C12 cells after treatment, we calculated the differentiation index as the percentage of MHC-positive cells above total nuclei and the fusion index as the average number of nuclei in MHC-positive cells with at least three nuclei above total number of nuclei, respectively.

### Quantitative real-time reverse transcription PCR

Total RNA (2 μg), extracted with TRI reagent from C2C12 myoblasts, was reverse transcribed using the high capacity cDNA reverse transcription kit (Applied Biosystems). The quantification of S1PR mRNA was performed by real-time PCR employing TaqMan gene expression assays. Each measurement was carried out in triplicate, using the automated ABI Prism 7700 Sequence Detector System (Applied Biosystems) as described previously [18], by simultaneous amplification of the target sequence (S1P_1_ Mm00514644_m1, S1P_2_ Mm01177794_m1, S1P_3_ Mm00515669_m1, and S1P_4_ Mm00468695_s1; Applied Biosystems) together with the housekeeping gene 18 S rRNA. Results were analyzed by ABI Prism Sequence Detection System software, version 1.7 (Applied Biosystems). The 2^-ΔΔCT^ method was applied as a comparative method of quantification [19], and data were normalized to ribosomal 18 S RNA expression.

### Cell proliferation

Cell proliferation was determined by measuring [^3^ H]Thymidine incorporation; C2C12 cells were serum-starved for 24 h and then challenged with or without 50 ng/ml IGF-1 for 16 h. [^3^ H]Thymidine (1 μCi/well) was added for the last 1 h of incubation. Cells were washed twice in ice-cold PBS before the addition of 500 μl of 10% trichloroacetic acid for 5 minutes at 4°C. Cells were washed again in ice-cold PBS, and 250 μl of ethanol:ether (3:1 v/v) was added to the insoluble material. Samples were then lysed in 0.25 N NaOH for 1 h at 37°C. Incorporation of [^3^ H]Thymidine was measured by scintillation counting.

### Statistical analysis

Statistical analysis was performed using Student’s *t* test. Graphical representations were performed using Prism 5.0 (GraphPad Software, San Diego, CA, USA). Densitometric analysis of the Western blot bands was performed using imaging and analysis software Quantity One (Bio-Rad Laboratories, Hercules, CA, USA).

## Results

### Insulin-like growth factor-1 stimulates sphingosine kinase in C2C12 myoblasts

**Figure 1:**
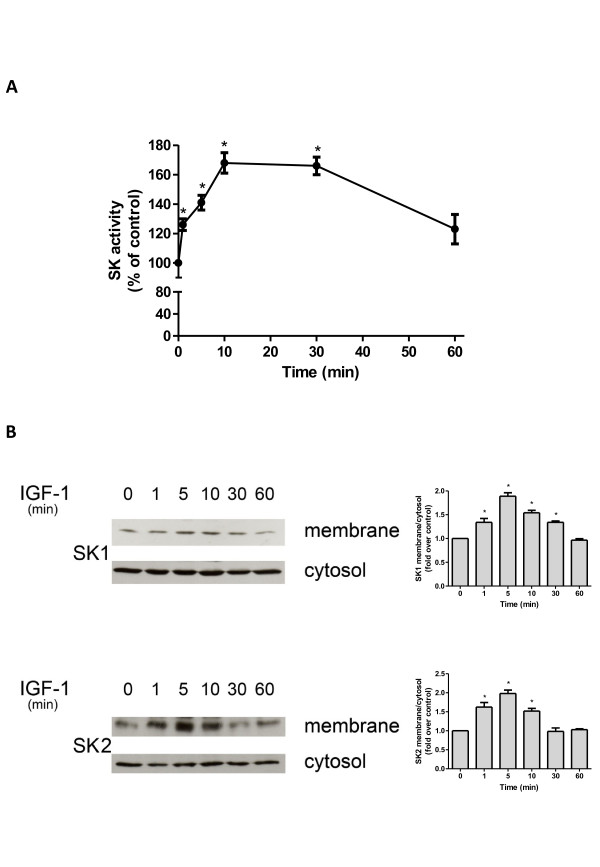
**Effect of insulin-like growth factor-1 on sphingosine kinase activity and subcellular localization.** Serum-starved myoblasts were incubated with 50 ng/ml insulin-like growth factor-1 (IGF-1) for the indicated time intervals. (**A**) Aliquots of cell extracts (40 μg) were used to determine sphingosine kinase (SK) activity. Data represent the mean ± SEM of at least three independent experiments, each performed at least in duplicate. The effect of IGF-1 in challenged versus unchallenged cells was statistically significant by Student’s *t* test (*P < 0.05). (**B**) Western analysis of SK1 (upper panel) and SK2 (lower panel) were performed in membrane and cytosolic fractions. Blots representative of at least three independent experiments are shown. The histograms represent densitometric analysis of three independent experiments. Data reported are expressed as -fold increase of the membrane:cytosol ratio. The increase of SK1 and SK2 membrane content induced by IGF-1 was statistically significant by Student’s *t*-test (*P < 0.05).

To explore whether the SK/S1P axis is involved in IGF-1 biological action, we first examined whether the growth factor was capable of regulating SK activity in C2C12 cells. Data illustrated in Figure 1A show that 50 ng/ml IGF-1 stimulated SK activity. Indeed, the growth factor rapidly and transiently increased SK activity, peaking at 10 minutes and returning to basal level at 60 minutes. In agreement, as shown in Figure 1B, myoblast treatment with 50 ng/ml IGF-1 was responsible for rapid and transient translocation of both enzyme isoforms, SK1 and SK2, to membrane fraction that was appreciable within 1 minute of incubation, reached the maximal effect at 5 minutes and declined thereafter, thus enhancing the amount of active form of the two enzymes with a favorable access to the hydrophobic substrate sphingosine.

**Figure 2:**
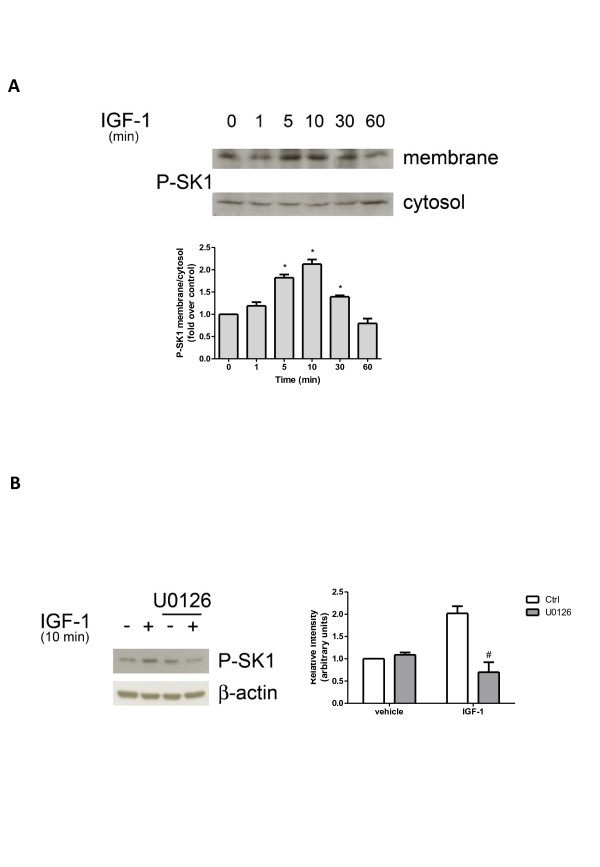
**Sphingosine kinase-1 phosphorylation induced by insulin-like growth factor-1 is mediated by ERK 1/2.** (**A**) Western blot analysis was performed using specific anti-phospho-sphingosine kinase (SK)1 antibody in membrane and cytosolic fractions prepared from serum-starved myoblasts treated with 50 ng/ml insulin-like growth factor-1 (IGF-1) for the indicated time intervals. A blot representative of at least three independent experiments with analogous results is shown. The histogram represents densitometric analysis of three independent experiments. Data reported are expressed as -fold increase of the membrane:cytosol ratio. The increase of phospho-SK1 membrane content induced by IGF-1 was statistically significant by Student’s *t* test (*P < 0.05). (**B**) Serum-starved myoblasts were pre-incubated for 30 minutes with ERK1/2 specific inhibitor (5 μM U0126) before being challenged with 50 ng/ml IGF-1 for 10 minutes. Cell lysates were separated by SDS-PAGE and immunoblotted using specific anti-phospho-SK1 and anti-β-actin antibodies. A blot representative of at least three independent experiments is shown. In the histogram band intensity corresponding to phosphorylated SK1 was normalized to β-actin and reported as mean ± SEM of three independent experiments, -fold change over control (set as 1). SK1 phosphorylation in IGF-1-challenged cells was significantly reduced by U0126 pre-incubation (Student’s *t* test, #*P* < 0.05).

Since agonist-induced stimulation of SK1 activity and translocation of the enzyme to the plasma membrane appears to be mediated by ERK1/2 phosphorylation at Ser^225^[15], Western blot analysis using a specific anti-phospho-SK1 antibody was performed in myoblast subcellular fractions following IGF-1 treatment. Data reported in Figure 2A show that cell challenge with 50 ng/ml IGF-1 resulted in a rapid increase in the phosphorylation of membrane-associated SK1, already detectable at 5 minutes with a maximum at 10 minutes of incubation. Interestingly, the kinetic of membrane translocation of phospho-SK1 was consistent with the time-course of enzyme activation induced by IGF-1 (Figure 1). Given the ability of IGF-1 to activate the ERK1/2 signaling pathway through its receptor, we analyzed the involvement of this pathway in the activation of SK1 induced by the growth factor. For this purpose, cells were treated with 5 μM U0126, a specific pharmacological inhibitor of the ERK1/2 pathway, 30 minutes before IGF-1 challenge. Results presented in Figure 2B show that the inhibition of the ERK1/2 pathway prevented SK1 phosphorylation induced by 10 minutes treatment with IGF-1, demonstrating that the activation of SK1 induced by the growth factor was mediated by ERK1/2.

**Figure 3:**
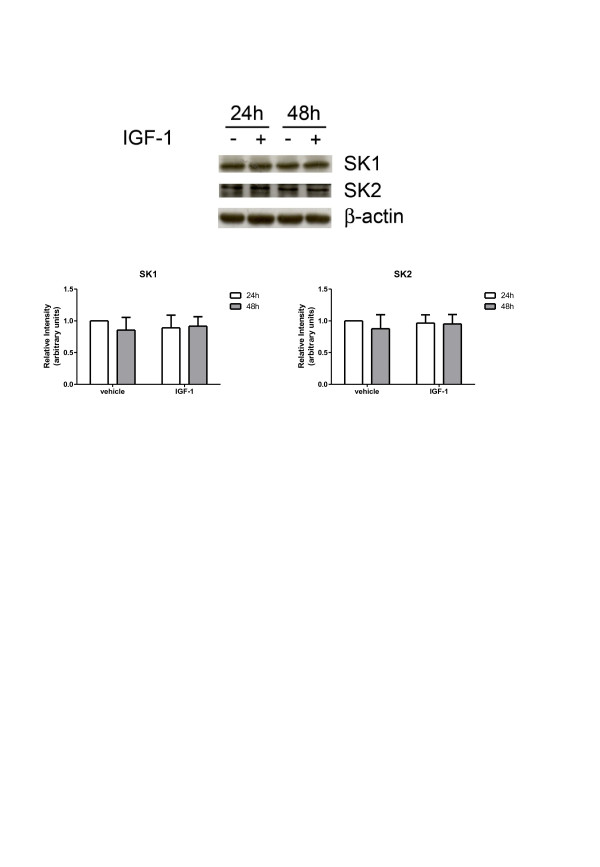
**Insulin-like growth factor-1 does not affect sphingosine kinase-1 or sphingosine kinase-2 protein content.** C2C12 myoblasts were incubated with DMEM containing 1 mg/ml bovine serum albumin (BSA) for the indicated time intervals in the absence (−) or in the presence (+) of 50 ng/ml insulin-like growth factor-1 (IGF-1). Top, aliquots of total cell lysates were used to perform Western analysis, using specific anti-sphingosine kinase (SK)1 and anti-SK2 antibodies. A representative blot is shown. Bottom, densitometric analysis of at least three independent experiments. Data are the mean ± SEM and are reported as protein expression normalized to β-actin, -fold change over control (set as 1).

We then investigated whether IGF-1 was also able to regulate the protein content of SK1 and SK2. As illustrated in Figure 3, Western blot analysis shows that myoblast treatment with 50 ng/ml IGF-1 for 24 h or 48 h did not affect SK1 or SK2 protein levels, ruling out this possibility.

### Involvement of sphingosine kinase in the myogenic effect of insulin-like growth factor-1

**Figure 4:**
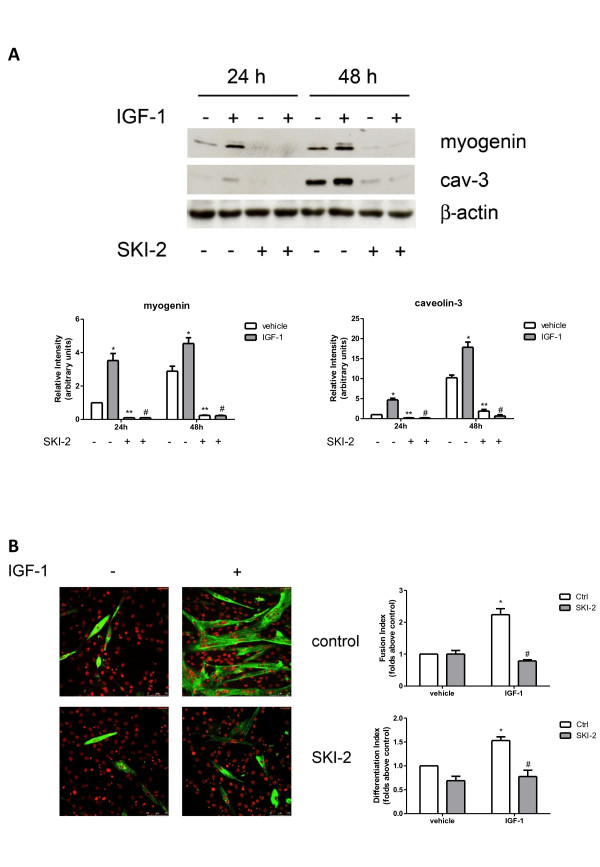
**The pro-myogenic effect of insulin-like growth factor-1 depends on sphingosine kinase engagement.** (**A**) C2C12 myoblasts were pre-incubated for 30 minutes with sphingosine kinase (SK) inhibitor (10 μM SKI-2) before being challenged with 50 ng/ml insulin-like growth factor (IGF-1) for the indicated time intervals. The content of myogenin and caveolin-3 (cav-3) was analyzed by Western blotting in cell lysates. Equally loaded protein was checked by expression of the non-muscle-specific β isoform of actin. A blot representative of four independent experiments with analogous results is shown. The histograms represent band intensity of myogenin and cav-3 normalized to β-actin and reported as mean ± SEM of four independent experiments, -fold change over control (time 24 h, no addition; set as 1). The effect of IGF-1 in challenged versus unchallenged cells at 24 h and 48 h on the expression levels of the myogenic markers was statistically significant by Student’s *t* test (*P < 0.05). The effect of SK inhibition on basal expression levels of the myogenic markers in SKI-2 treated versus SKI-2 untreated cells was statistically significant by Student’s *t* test (**P < 0.05). The effect of SK inhibition on IGF-1-induced expression of myogenin and cav-3 in challenged cells (IGF-1 and SKI-2 added) versus control (IGF-1 added, no SKI-2) was statistically significant by Student’s *t* test (#*P* < 0.05). (**B**) C2C12 cells were seeded on microscope slides and treated as described in A. Representative immunofluorescence images of C2C12 myoblasts treated for 72 h with 50 ng/ml IGF-1 stained with anti-myosin heavy chain (MHC) antibody and propidium iodide are shown. Fusion index and differentiation index were calculated as described in the Methods section. Data are mean ± SEM of three independent experiments. The effect of IGF-1 in challenged versus unchallenged cells on fusion index and differentiation index was statistically significant by Student’s *t* test (*P < 0.05); fusion index and differentiation index in IGF-1-challenged cells were significantly reduced by SKI-2 pre-incubation (Student’s *t* test #*P* < 0.05).

**Figure 5:**
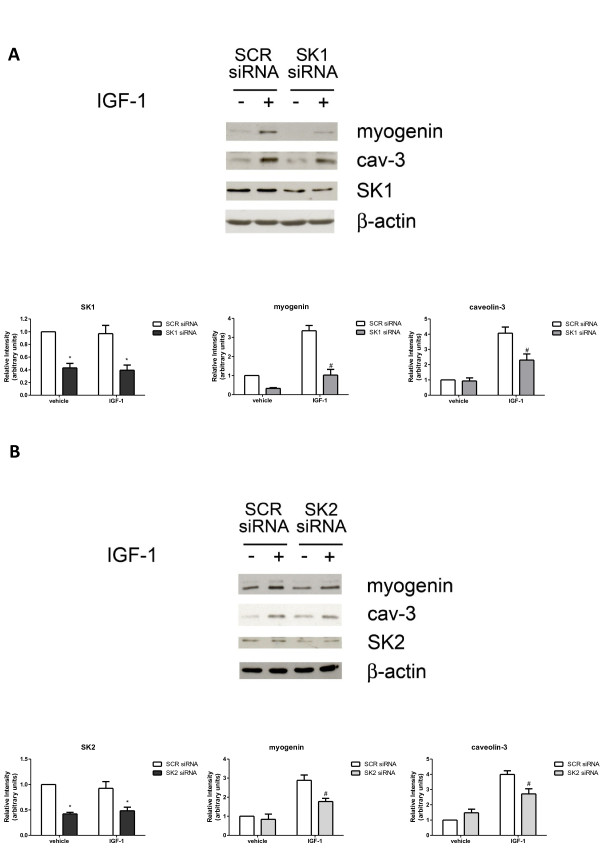
**The pro-myogenic effect of insulin-like growth factor-1 depends on sphingosine kinase-1 and sphingosine kinase-2 engagement.** (**A**) C2C12 myoblasts, transfected with scrambled (SCR) or sphingosine kinase (SK)1-siRNA were challenged with 50 ng/ml insulin-like growth factor-1 (IGF-1) for the last 24 h of transfection. Western analysis of SK1 and skeletal muscle marker proteins were performed in cell lysates. Equally loaded protein was checked by expression of the non-muscle-specific β isoform of actin. A blot representative of three independent experiments with analogous results is shown. The histograms represent band intensity of SK1, myogenin and caveolin-3 (cav-3) normalized to β-actin and reported as mean ± SEM of three independent experiments, -fold change over control (time 24 h, no addition; set as 1). (**B**) Myoblasts transfected with SCR- or SK2-siRNA were treated and used as described in A. SK silencing in SK1- and SK2-siRNA transfected cells versus SCR-siRNA transfected cells was statistically significant (*P < 0.05). The effect of SK downregulation on IGF-1-induced expression of myogenin and caveolin-3 in IGF-1-challenged, SK-siRNA transfected cells versus control (IGF-1 added, SCR-siRNA transfection) was statistically significant by Student’s *t* test (#*P* < 0.05).

To confirm the ability of IGF-1 to promote myoblast differentiation, the expression of skeletal muscle marker proteins such as myogenin and caveolin-3, normally absent in immature myoblasts but strongly expressed in differentiated cells, was determined by Western analysis. Results presented in Figure 4A show that the expression of myogenic markers in unchallenged serum-deprived myoblasts was enhanced in a time-dependent manner, in agreement with the release of autocrine differentiation factors [20,21]. As expected, myoblast treatment with 50 ng/ml IGF-1 strongly increased the expression of both the myogenic markers at 24 and 48 h. The possible involvement of SK in the myogenic effect of the growth factor was then investigated. To this aim, C2C12 myoblasts were treated with SKI-2, a specific pharmacological inhibitor of SK, already successfully used to block the enzymatic activity in these cells [10]. In agreement with the established role of the SK/S1P axis in myogenesis [7,22], 30 minutes pre-incubation with 10 μM SKI-2 significantly decreased the protein content of myogenin and caveolin-3 in unchallenged cells (Figure 4A). Interestingly, cell treatment with the inhibitor completely reversed the enhanced expression of the two myogenic markers induced by IGF-1 at all the examined time-intervals, suggesting a key role of SK in IGF-1-induced myogenesis. In keeping with its well established pro-myogenic action, IGF-1 (50 ng/ml) at 72 h incubation significantly augmented fusion index and differentiation index, but its biological response was completely prevented by previous incubation with SKI-2 (Figure 4B). These results demonstrate that the IGF-1-dependent myogenic process strictly depends on the activation of SK. To further assess the involvement of SK in the myogenic response evoked by IGF-1 in murine myoblasts, SK isoforms were specifically knocked-down by employing siRNA technology. In accordance with previous reports [10,22], specific siRNA treatment efficaciously reduced basal expression levels of SK1 (Figure 5A). The three-fold IGF-1-dependent increase of myogenin expression was totally abrogated when SK1 was downregulated by RNA interference, while caveolin-3 content, increased by the growth factor by approximately 3.5-fold, was robustly decreased (approximately two-fold) (Figure 5A). Furthermore, the myogenic effect of IGF-1 was also reduced when SK2 was downregulated by specific RNA interference (Figure 5B). Indeed, Figure 5B shows that silencing of SK2 expression blunted the increase of myogenin and caveolin-3 content elicited by 50 ng/ml IGF-1 by approximately 1.5-fold. The knocking down experiments support a role for both SK isoforms in the differentiation driven by IGF-1. Moreover, the reduced effectiveness of SK2 downregulation in comparison to SK1 in preventing the myogenic effect of the growth factor suggests that the SK1 isoform plays a dominant role.

### The pro-myogenic effect of insulin-like growth factor-1 depends on sphingosine 1-phosphate_2_ receptor engagement

**Figure 6:**
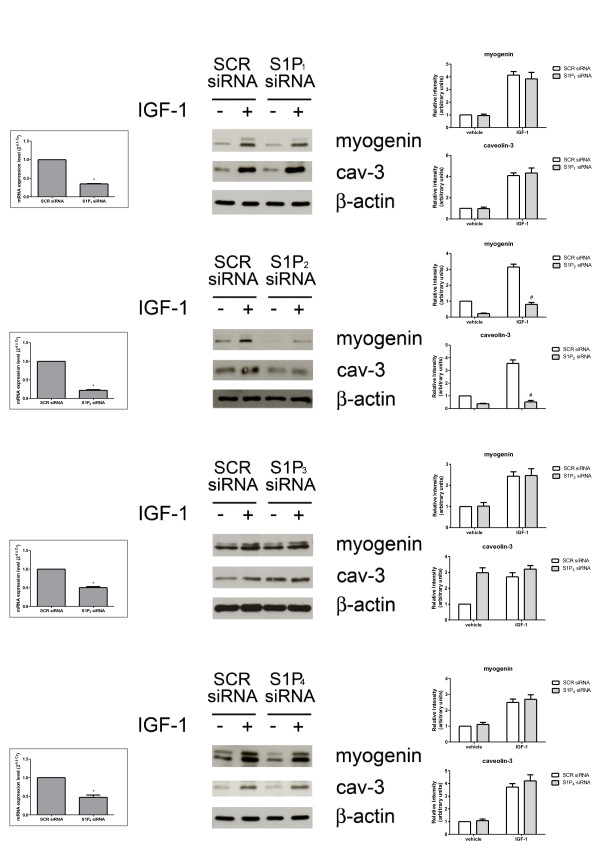
**Role of sphingosine 1-phosphate receptors in insulin-like growth factor-1-induced differentiation of mouse myoblasts.** C2C12 myoblasts, transfected with scrambled (SCR) or with specific siRNA for individual sphingosine 1-phosphate receptors (S1PR), incubated in the absence (−) or in the presence (+) of 50 ng/ml insulin-like growth factor-1 (IGF-1) for the last 24 h of transfection, were checked for downregulation by real-time PCR (left). Middle, Western analysis of skeletal muscle marker proteins was performed in cell lysates. Equally loaded protein was checked by expression of the non-muscle-specific β isoform of actin. A blot representative of three independent experiments with analogous results is shown. Right, densitometric analysis. The histograms represent band intensity of myogenin and caveolin-3 (cav-3) normalized to β-actin and reported as mean ± SEM of three independent experiments, -fold change over control (time 24 h, no addition; set as 1). The effect of S1P_2_ downregulation on IGF-1-induced expression of myogenin and cav-3 in IGF-1-challenged, S1P_2_-siRNA transfected cells versus IGF-1-challenged, SCR-siRNA transfected cells was statistically significant by Student’s *t* test (#*P* < 0.05); S1PR silencing was statistically significant in S1PR-siRNA transfected cells versus SCR-transfected cells (*P < 0.05).

We have previously demonstrated that exogenous S1P exerts pro-myogenic action in C2C12 cells via engagement of the S1P_2_ receptor [7]. Since IGF-1 is able to increase S1P biosynthesis by activating both SK1 and SK2, and given that this event is crucial for the differentiating action of the growth factor, we investigated whether the myogenic action exerted by IGF-1 was mediated by S1P_2_ engagement in myoblasts. For this purpose, the expression of skeletal muscle marker proteins induced by 50 ng/ml IGF-1 was evaluated in myoblasts where S1P_2_ was specifically downregulated by RNA interference. As shown in Figure 6, siRNA directed against S1P_2_, which significantly reduced the receptor expression, strongly attenuated the enhancement of myogenin elicited by IGF-1 (approximately three-fold) and abrogated the increase of caveolin-3. Conversely, the downregulation of the other receptor isoforms, S1P_1_, S1P_3_ or S1P_4_, by specific siRNA did not alter the myogenic effect of the growth factor (Figure 6). The observed appreciable stimulation of the expression of caveolin-3 in unchallenged cells where S1P_3_ was downregulated (Figure 6) was in agreement with the previously demonstrated anti-myogenic action mediated by this receptor subtype [7]. Altogether, these data support the view that transactivation of S1P_2_ by IGF-1 is implicated in the myogenic effect of the growth factor in myoblasts.

### Sphingosine kinase/sphingosine 1-phosphate signaling pathway is negatively implicated in the mitogenic effect of insulin-like growth factor-1

**Figure 7:**
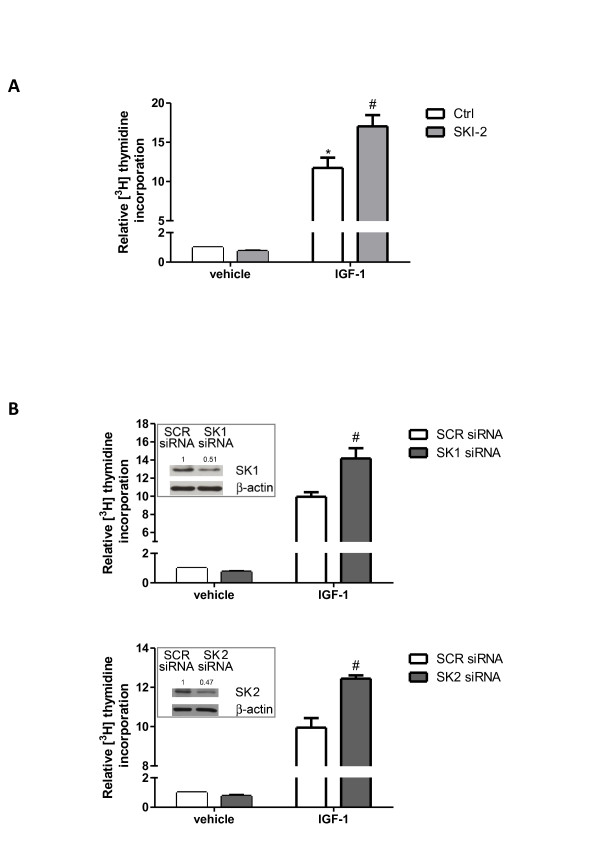
**Role of sphingosine kinase in the mitogenic effect of insulin-like growth factor-1.** (**A**) Serum-starved C2C12 myoblasts were pre-incubated for 30 min in the presence or absence of 1 μM SKI-2 before being stimulated with 50 ng/ml insulin-like growth factor-1 (IGF-1) for 16 h. [^3^ H]Thymidine (1 μCi/well) was added during the last hour of incubation. [^3^ H]Thymidine incorporation in untreated cells was 29893 ± 1584 dpm. Results are reported as -fold change over control (vehicle, no addition; set as 1). Data are mean ± SEM of at least three independent experiments performed in triplicate. The mitogenic effect of IGF-1 in challenged versus unchallenged cells was statistically significant by Student’s *t* test (*P < 0.05). The effect of SK inhibition on the mitogenic effect of IGF-1 in challenged cells versus control (no SKI-2, IGF-1 added) was statistically significant by Student’s *t* test (#*P* < 0.05). (**B**) Scrambled (SCR) or specific SK1- (upper panel) or SK2-siRNA (lower panel) transfected C2C12 cells were treated or not treated with 50 ng/ml IGF-1 for 16 h. [^3^ H]Thymidine (1 μCi/well) was added during the last hour of incubation. [^3^ H]Thymidine incorporation was 20781 ± 785 dpm in control cells (untreated SCR-transfected cells). Results are reported as -fold change over control (SCR siRNA, no addition; set as 1). Data are mean ± SEM of at least four independent experiments performed in triplicate. The effect of SK downregulation on IGF-1-induced [^3^ H]Thymidine incorporation in IGF-1-challenged, SK1- and SK2-siRNA transfected cells versus control (IGF-1 added, SCR-siRNA transfection) was statistically significant by Student's *t* test (#*P* < 0.05). Insert: cell extracts from C2C12 myoblasts transfected with SCR-, SK1-siRNA or SK2-siRNA were employed for Western analysis using anti-SK1 or anti-SK2 antibodies. Equally loaded protein was checked by expression of β-actin. A blot representative of at least four independent experiments with analogous results is shown. Band intensity of SK1 or SK2 was normalized to β-actin and reported as -fold change over control (SCR siRNA, no addition; set as 1).

Besides stimulating myogenesis, IGF-1 is also critically implicated in the control of myoblast growth. Therefore, we investigated whether the activation of the SK/S1P signaling pathway mediated by IGF-1 was also implicated in the mitogenic effect of the growth factor. Data presented in Figure 7A show that 50 ng/ml IGF-1 strongly stimulated cell proliferation, determined by measuring the incorporation of ^3^ H] Thymidine into DNA, confirming the reported mitogenic effect of the growth factor in these cells [12]. Interestingly, when myoblasts were previously incubated with the specific pharmacological inhibitor of SK (SKI-2), the mitogenic effect of IGF-1 was significantly enhanced, suggesting a negative role of SK in modulating the proliferative response elicited by the growth factor. To further support this finding, individual SK isoforms were knocked-down by specific siRNA treatment (Figure 7B, insert). In accordance with the results obtained employing the pharmacological inhibitor SKI-2, data presented in Figure 7B demonstrate that knocking-down of SK1, as well as SK2, strongly increased the mitogenic effect elicited by IGF-1 by 90% and 50%, respectively.

**Figure 8:**
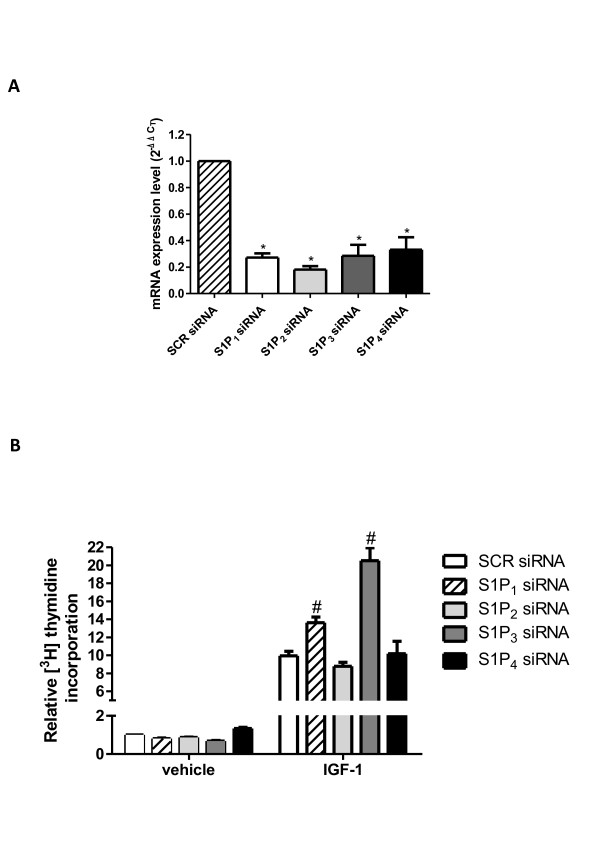
**Role of sphingosine 1-phosphate receptors in the mitogenic effect of insulin-like growth factor-1.** (**A**) Quantitative mRNA analysis was performed in C2C12 myoblasts transfected with non-specific scrambled (SCR) siRNA or with siRNA specific for sphingosine 1-phosphate receptors (S1PR); the content of housekeeping gene 18 S rRNA was analyzed in parallel. Results are expressed as -fold changes according to the 2^−ΔΔCT^ method, utilizing each receptor subtype in cells transfected with SCR-siRNA as a calibrator. Data are mean ± SEM of three independent experiments performed in triplicate. S1PR silencing in S1PR-siRNA transfected cells versus SCR-siRNA transfected cells was statistically significant (*P < 0.05). (**B**) Serum-starved C2C12 myoblasts transfected with unspecific SCR-siRNA, or with specific siRNA for individual S1PR, were stimulated with 50 ng/ml insulin-like growth factor 1 (IGF-1) for 16 h. [^3^ H]Thymidine (1 μCi/well) was added during the last hour of incubation. [^3^ H]Thymidine incorporation was 19643 ± 844 dpm in control cells (untreated SCR-transfected cells). Results are reported as -fold change over control (SCR siRNA, no addition; set as 1). Data are mean ± SEM of at least three independent experiments performed in triplicate. The effect of S1P_1_ and S1P_3_ downregulation on the IGF-1-induced [^3^H]Thymidine incorporation in IGF-1-challenged, S1P_1_- and S1P_3_-siRNA transfected cells versus IGF-1-challenged, SCR-siRNA transfected cells was statistically significant by Student's *t* test (#*P* < 0.05).

To establish whether the mechanism by which the SK/S1P axis inhibits IGF-1-induced cell proliferation relies on receptor engagement, labelled thymidine incorporation experiments were performed in cells where individual S1PR were specifically knocked down by siRNA technology (Figure 8A). Results illustrated in Figure 8B clearly show that downregulation of S1P_1_ or S1P_3_ by RNA interference significantly enhanced DNA synthesis induced by 50 ng/ml IGF-1, whereas cell transfection with siRNA that specifically downregulated S1P_2_ or S1P_4_ did not impair the mitogenic effect of the growth factor. These data demonstrate that enhanced biosynthesis of S1P, due to SK1 and SK2 activation induced by IGF-1, is responsible for the engagement of S1P_1_ and S1P_3_ and is negatively implicated in the mitogenic effect of the growth factor.

### Sphingosine 1-phosphate lyase is not implicated in the insulin-like growth factor-1 biological role in skeletal muscle

**Figure 9:**
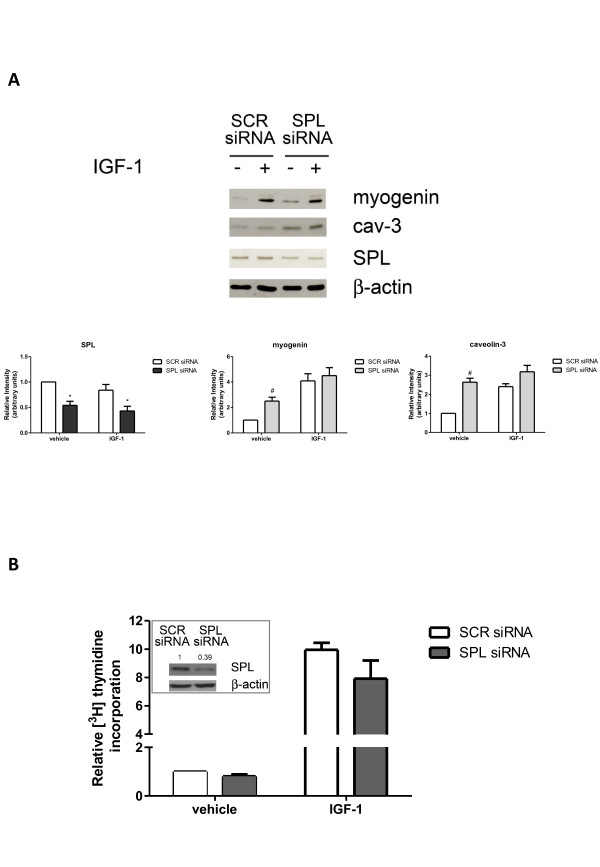
**Sphingosine 1-phosphate lyase is not involved in insulin-like growth factor-1 biological action in C2C12 myoblasts.** (**A**) C2C12 myoblasts, transfected with scrambled (SCR) or sphingosine 1-phosphate lyase (SPL)-siRNA, were incubated in the absence (−) or in the presence (+) of 50 ng/ml insulin-like growth factor-1 (IGF-1) for the last 24 h of transfection. Western analysis of SPL and skeletal muscle marker proteins was performed in cell lysates. Equally loaded protein was checked by expression of the non-muscle-specific β isoform of actin. A blot representative of three independent experiments with analogous results is shown. The histograms represent band intensity of SPL, myogenin and caveolin-3 (cav-3) normalized to β-actin and reported as mean ± SEM of three independent experiments, -fold change over control (time 24 h, no addition; set as 1). SPL silencing in SPL-siRNA transfected cells versus SCR-siRNA transfected cells was statistically significant by Student’s *t* test (*P < 0.05). The effect of SPL downregulation on basal expression levels of myogenin and cav-3 in SPL-siRNA transfected, unchallenged cells versus SCR-siRNA transfected, unchallenged cells was statistically significant by Student’s *t* test (#*P* < 0.05). (**B**) Myoblasts transfected with unspecific SCR-siRNA or with specific SPL-siRNA were treated or not treated with 50 ng/ml IGF-1 for 16 h. [^3^ H]Thymidine (1 μCi/well) was added during the last hour of incubation. [^3^ H]Thymidine incorporation was 21781 ± 993 dpm in control cells (untreated SCR-transfected cells). Results are reported as -fold change over control (SCR siRNA, no addition; set as 1). Data are mean ± SEM of at least three independent experiments performed in triplicate. Insert: cell extracts from C2C12 myoblasts transfected with SCR- and SPL-siRNA were employed for Western analysis using anti-SPL antibodies. Equally loaded protein was checked by expression of β-actin. A blot representative of at least four independent experiments with analogous results is shown. Band intensity of SPL was normalized to β-actin and reported as -fold change over control (SCR siRNA, no addition; set as 1).

Finally, since cell response to IGF-1 was found to depend on S1P intracellular levels, we investigated whether SPL, responsible for the irreversible degradation of S1P, is involved in the biological effect of the growth factor. Western analysis data presented in Figure 9A show that treatment with 50 ng/ml IGF-1 did not affect SPL protein content in C2C12 myoblasts. In the same figure it is shown that the increased expression of skeletal muscle marker proteins induced by 50 ng/ml IGF-1 was not affected by specific downregulation of SPL by RNA interference, ruling out the involvement of this enzyme in the control of the IGF-1 myogenic effect. It is interesting to note that knocking-down of SPL enhanced basal expression levels of the myogenic markers by approximately two-fold, in favor of the participation of the enzyme in the regulation of intracellular levels of S1P, whose myogenic effect has been previously demonstrated [7,22].

We then investigated the role of SPL in IGF-1-induced DNA synthesis. For this purpose, labelled thymidine incorporation experiments were performed in cells where SPL had been specifically downregulated by employing siRNA technology. Data presented in Figure 9B clearly show that cell transfection with siRNA that specifically downregulated SPL (Figure 9B, insert) did not impair the mitogenic effect of IGF-1, demonstrating that SPL does not participate in the IGF-1-directed regulation of S1P metabolism.

## Discussion

The IGF-1 signaling pathway plays a key role in the regulation of skeletal muscle growth, in differentiation, and in the maintaining of tissue homeostasis. Intriguingly, this growth factor stimulates two key processes such as myoblast proliferation and differentiation which are mutually exclusive events during myogenesis [12]. The results presented here for the first time demonstrate that the SK/S1P signaling pathway is involved in the regulation of the biological action exerted by IGF-1. This finding is crucial for the timely regulation of cell proliferation and differentiation. Indeed, experimental evidence is provided that activation of SK followed by transactivation of S1P_2_ elicited by the growth factor is required for its pro-myogenic action whereas the parallel engagement of S1P_1_ and S1P_3_ reduces its mitogenic effect. Notably, the IGF1-induced SK/S1P axis appears to exhibit the unique property of regulating two opposite biological effects elicited by IGF-1 in myoblasts - transducing its myogenic response on one side and inhibiting its mitogenic effect on the other - therefore acting as an efficacious switch to stop proliferation and triggering differentiation through the concomitant activation of individual S1PR subtypes.

Previously we have demonstrated in these cells that the final biological outcome of PDGF, which plays a crucial role in skeletal muscle, depends on SK/S1P axis activation [11]. Indeed, the activation of SK1 induced by PDGF resulted in the transactivation of S1P_1_, responsible for the chemotactic response elicited by PDGF and also for the negative regulation of its mitogenic effect. Despite the overlapping role of the SK/S1P axis in inhibiting the mitogenic effect of both PDGF and IGF-1, we show here that the negative regulation of IGF-1-induced proliferation, beside being dependent on S1P_1_, relies also on S1P_3_ engagement. Moreover, in this study, we have established that the IGF-1 myogenic effect is dependent on the engagement of S1P_2_, which has been already shown to be coupled to the pro-differentiating activity of exogenous and endogenous S1P in these cells [7,22].

Interestingly, here we provide the first evidence of the involvement of SK2 in S1P inside-out signaling in myoblasts. Indeed SK2, similarly to the SK1 isoform, was found to be required for transmitting the pro-myogenic effect of IGF-1, highlighting a key role of this enzyme isoform in skeletal muscle biology. In this regard, the parallel regulation of mitogenesis and migration elicited by PDGF was found to rely exclusively on the specific activation of SK1 [11]. SK1 and SK2 have different developmental expression, adult tissue distribution and subcellular localization patterns. Such differences are in line with the concept that, although the two enzymes use the same substrate and generate the same product, they can mediate distinct biological events. SK1 is almost universally associated with the induction of cell survival and proliferation [23], whereas SK2 overexpression suppresses cell growth and enhances apoptosis [24]. Studies performed in mesangial cells supported the pro-apoptotic role of SK2, demonstrating that SK2-null cells are more resistant to staurosporine-induced apoptosis than wild-type or SK1-null cells [25]. Our findings corroborate the notion that SK isoforms may instead have overlapping functions. Indeed, data obtained by preincubation with the pharmacological inhibitor SKI-2 that does not distinguish between the two SK isoforms, and by specific silencing of SK1 or SK2 isoforms demonstrate the involvement of both enzyme isoforms in the pro-myogenic effect of IGF-1. In accordance, it has been recently reported that SK1 and SK2 are equally required for epidermal growth factor-induced migration of breast cancer cells [26] and TGFβ-induced migration and invasion of esophageal cancer cells [27].

Data previously reported in the literature sustain the existence of a functional cross-talk between IGF-1 and S1P signaling pathways. IGF-1 was shown to elicit ERK1/2 activation by stimulating SK1-dependent transactivation of S1P_1_ even though the biological effect of S1P-dependent ERK1/2 activation was not addressed [28,29]. In this study, for the first time, we identified the involvement of the SK/S1PR axis in the biological action of IGF-1, that intriguingly in its molecular mechanism of action implies ERK1/2-dependent phosphorylation of SK1.

It is worth noting that exogenous S1P has been previously reported to counteract the chemotactic action exerted by IGF-1 in myoblasts via ligation of S1P_2_[8]. The involvement reported in this paper of the SK/S1P axis in mediating IGF-1 biological effects is not in constrast with the previous finding. Indeed, it appears that the selective spatial control of S1P formation inside myoblasts following stimulation with different cues is crucial for the generation of a specific biochemical response. In this regard, we recently demonstrated that endogenous S1P that is formed in response to PDGF challenge stimulates cell motility via S1P_1_[11], whereas exogenous S1P was previously found to inhibit motility of the same cell type acting through S1P_2_[8]. Moreover, we recently demonstrated that, in C2C12 myoblasts, the S1P signaling pathway, which physiologically accounts for myogenic differentiation via S1P_2_, is redirected by the cytokine TGFβ to act primarily via the pro-fibrotic S1P_3_. Indeed, consequently to TGFβ treatment, S1P_3_ then becomes the prevailing expressed receptor, thus promoting myoblast transdifferentiation to myofibroblasts [9].

Finally, we investigate the possible involvement of SPL in skeletal muscle biology. A previous study performed by Herr and colleagues [30] demonstrated that normal S1P catabolism is required for Drosophila muscle development, given that SPL-null mutants exhibited pattern abnormalities in the dorsal longitudinal flight muscles of the adult thorax. Moreover, SPL was recently identified as a potential therapeutic target for ischemia-reperfusion injury of the heart [31]. Using a genetic SPL knock-out mouse model and a chemical inhibitor, Bandhuvula and colleagues [31] demonstrated that ischemia caused the activation of SPL in cardiac tissue resulting in the reduction of the levels of S1P, thus promoting cardiomyocyte apoptosis. For the first time, we have demonstrated in this paper that mouse myoblasts express SPL. Furthermore, we provide evidence that this enzyme contributes to the regulation of intracellular levels of the pro-myogenic S1P, as knocking-down of the enzyme resulted in the upregulation of the basal expression levels of myogenic markers. This is in agreement with the concept that, in this experimental condition, S1P intracellular levels are enhanced and consequently the mediated-promyogenic action is potentiated [7,22]. However, from this study, SPL appears to be disengaged from IGF-1-mediated biological effects as its specific downregulation did not affect either myogenesis or proliferation induced by the growth factor.

Taking into account the different S1PR patterns expressed in satellite cells and murine myoblasts, and considering that exogenous S1P is mitogenic in satellite cells whereas it acts as an anti-mitogenic and pro-differentiating cue in myoblasts [6,7], it will be interesting to further investigate whether the individuated cross-talk between the IGF-1 and S1P signaling pathway observed here also takes place in satellite cells and mediates a specific IGF-1-induced biological response

## Conclusions

Collectively, our findings support the notion that the SK/S1P axis, via the engagement of S1PR, exhibits the unique ability to regulate two opposite biological effects elicited by IGF-1 in myoblasts - transducing its myogenic response on one side and inhibiting its mitogenic effect on the other. These results increase our knowledge on the mechanism by which IGF-1 regulates skeletal muscle regeneration.

## Abbreviations

AEBSF, 4-(2-aminoethyl)benzenesulfonyl fluoride; BSA, bovine serum albumin; DMEM, Dulbecco’s modified Eagle’s medium; IGF-1, Insulin-like growth factor-1; MHC, myosin heavy chain; PBS, phosphate-buffered saline; PCR, polymerase chain reaction; PDGF, platelet-derived growth factor; S1P, sphingosine 1-phosphate; S1PR, sphingosine 1-phosphate receptors; SK, sphingosine kinase; SPL, sphingosine 1-phosphate lyase; TGF, transforming growth factor; TNF, tumor necrosis factor.

## Competing interests

The authors declare that they have no competing interests.

## Authors’ contributions

CB performed the experiments, analyzed the data and wrote the article. FC performed the experiments and analyzed the data. SB performed the experiments. CD analyzed the data and wrote the article. PB designed the research and wrote the article. All the authors read and approved the final manuscript.
